# Medical Teaching

**Published:** 1879-10

**Authors:** 


### MEDICAL TEACHING.

The season for medical lectures is now at hand, and the various institutions of the country are rubbing up for the winter session. The advantages to be found at each have been presented to the public throughout the various usual channels for several months past, giving students of medicine a fair opportunity to select an Ala Mater suited to their taste.

While New York and Philadelphia are sending out their numerous college circulars, Atlanta is coming forward with her claim to notice as a center also of diversified meadical teaching. Interchange of students with the North, West and South, is of course to be expected, but it can no longer be said that Atlanta does not afford thorough and abundant medical teaching. The indications are that more than the usual number of students will take their course of study in Atlanta the coming winter.

